# Characterization of HLA-G Regulation and HLA Expression in Breast Cancer and Malignant Melanoma Cell Lines upon IFN-γ Stimulation and Inhibition of DNA Methylation

**DOI:** 10.3390/ijms21124307

**Published:** 2020-06-17

**Authors:** Nanna Jørgensen, Abid Sayed, Helene Bjerregaard Jeppesen, Gry Persson, Iben Weisdorf, Tina Funck, Thomas Vauvert Faurschou Hviid

**Affiliations:** 1 Department of Clinical Biochemistry, Centre for Immune Regulation and Reproductive Immunology (CIRRI), The ReproHealth Consortium ZUH, Zealand University Hospital, 4000 Roskilde, Denmark; abidsayed2011@live.dk (A.S.); heleje@regionsjaelland.dk (H.B.J.); gryp@regionsjaelland.dk (G.P.); iber@regionsjaelland.dk (I.W.); tioh@regionsjaelland.dk (T.F.); 2 Department of Clinical Medicine, University of Copenhagen, 2200 Copenhagen N, Denmark; 3 Department of Science and Environment, Roskilde University, 4000 Roskilde, Denmark

**Keywords:** HLA-G, breast cancer, malignant melanoma, in vitro modification, DNA methylation, immunoediting

## Abstract

The potential role of human leukocyte antigen (HLA)-G as a target for new cancer immunotherapy drugs has increased the interest in the analysis of mechanisms by which HLA-G expression is regulated, and how the expression can be manipulated. We characterized HLA expression in breast cancer and malignant melanoma cell lines and investigated the induction of HLA-G expression by two distinct mechanisms: stimulation with interferon (IFN)-γ or inhibition of methylation by treatment with 5-aza-2’-deoxycytidine (5-aza-dC). The effect of IFN-γ and 5-aza-dC on HLA expression was dependent on the cancer cell lines studied. However, in general, surface expression of HLA class Ia was induced on all cell lines. Surface expression of HLA-G was inconclusive but induction of HLA-G mRNA was prevalent upon treatment with 5-aza-dC and a combination of IFN-γ and 5-aza-dC. IFN-γ alone failed to induce HLA-G expression in the HLA-G-negative cell lines. The results support that HLA-G expression is regulated partly by DNA methylation. Furthermore, IFN-γ may play a role in the maintenance of HLA-G expression rather than inducing expression. The study demonstrates the feasibility of manipulating HLA expression and contributes to the exploration of mechanisms that can be potential targets for immunotherapy in breast cancer and malignant melanoma.

## 1. Introduction

The assembly of immune responses is highly dependent on human leukocyte antigen (HLA) molecules. The HLA molecules are encoded in the human major histocompatibility complex (MHC) and mediate immune activation and immune suppression through HLA class Ia and class Ib molecules, respectively. The extent of immune regulation is inevitable in the context of cancer development and treatment and so is the role of HLA-mediated interaction between tumor and immune cells. Progression of cancer is often associated with altered expression of HLA molecules and multiple studies support that upregulation of HLA class Ib expression, especially HLA-G, is a mechanism, whereby tumors are able to escape immunological surveillance [1]. A concomitant loss or downregulation of HLA class Ia molecules can further enhance the ability of cancer cells to escape T cell recognition [2].

The highly polymorphic *HLA class Ia* genes, *HLA-A*, *-B*, and *-C*, are expressed on almost all nucleated cells. Whereas HLA class Ib mRNA expression is frequent in many tissues, surface expression is restricted in healthy tissue. Expression of HLA-G is mainly restricted to the trophoblast cells in the placenta, where it participates in maintaining tolerance at the fetal-maternal interface [3,4]. However, HLA-G expression is often induced in pathological settings and is frequently seen in various cancers [5]. Of the HLA class Ib molecules, HLA-G is probably the most studied in terms of cancer immunology and the most well-defined immunosuppressive molecule of the HLAs favoring tumor immune escape. Whereas knowledge of the role of HLA-F in cancer is sparse, HLA-E expression is also seen in several cases of cancers associated with immune suppression.

We have previously shown an association between high HLA-G and HLA-ABC expression with tumor aggressiveness and a worse prognosis in a study including 250 malignant melanoma patients [6]. Furthermore, HLA-G might serve as a prognostic marker in combination with HLA-E [7]. Expression of HLA-E is correlated with reduced immunogenicity in renal cell carcinoma [8], and in non-small cell lung carcinoma HLA-E expression is suggested to restrain the positive prognostic effect of infiltrating cytotoxic T cells [9]. A concomitant loss of HLA class Ia and high expression of HLA-G and HLA-E is associated with a worse prognosis and increased metastatic capacity in breast cancer [10,11,12]. Furthermore, both HLA-G and HLA-E expression are correlated with poor survival and tumor metastasis in colorectal cancer [13,14]. Interestingly, different HLA-G isoforms seem to differentially increase the surface expression of HLA-E [15].

HLA-G exist in seven well-recognized isoforms. HLA-G1 to -G4 are membrane-bound molecules and HLA-G5- to -G7 are soluble proteins due to alternative splicing and skipping of different exons [16,17,18,19]. Expression of the different isoforms might have different functional implications and even though the expression pattern of the HLA-G isoforms is controversial, some original in vitro studies indicate that the isoforms can substitute each other and have the same capacity to inhibit cytotoxic immune cells [20,21]. Furthermore, a recent study has identified a novel landscape of HLA-G isoforms in renal cell carcinoma patients [22]. Giving that the study reports a marked heterogeneity of the HLA-G expression, the authors emphasize how an extensive portrait of the HLA-G isoform profile can be favorable in development of future treatment strategies. Expression of HLA-G can be further affected by a 14 bp insertion/deletion (ins/del) polymorphism in exon 8, the 3′-untranslated region (UTR) of the HLA-G transcript [23]. This polymorphism influences both mRNA stability and isoform splice patterns. Some studies point towards HLA-G mRNA isoforms including the 14 bp sequence in the 3′UTR might be expressed at a lower level than corresponding isoforms with the 14 bp sequence deleted [24,25].

The inhibitory role of HLA-G contributes to the idea of HLA-G being an immune checkpoint molecule with therapeutic potential [26]. Current immunotherapeutic treatment options are already exploiting the interactions of other immune checkpoint inhibitory receptors with their respective ligands, such as program cell death protein 1 (PD-1) and PD-L1, and cytotoxic cell lymphocyte antigen 4 (CTLA-4) and CD80/CD86. Since these checkpoint molecules are targets for successful immunotherapy of selected cancers only, it is essential to study the molecular role and function of HLA-G as a possible target for new drugs.

Interestingly, HLA-G expression is rarely found on in vitro established cancer cell lines. Most likely, the tumor microenvironment plays a vital role in the induction of HLA-G and investigation of the specific mechanisms of differential HLA-G expression are highly important. Human interferon (IFN)-γ plays a role in tumor immunogenicity widely known as an anti-tumor cytokine but also with a dual role in immune evasion and tumor growth, for example through upregulation of the checkpoint inhibitor PD-L1 and CTLA-4 [27,28,29,30]. Previous studies have shown that IFN-γ is able to induce HLA-G expression in a number of cancer cell lines, and that the isoform profile can be regulated by the cytokine environment [31,32]. However, cytokine induced expression of HLA-G seems to be heterogeneous and highly dependent on the cell lineage. Furthermore, previous studies show that HLA-G expression can be induced in different tumor cell lines and mesenchymal stem cells by treatment with the demethylating agent 5-aza-2′-deoxycytidine (5-aza-dC) indicating that HLA-G expression is regulated partly by methylation-mediated repression [33,34,35].

A detailed molecular investigation of HLA-G and its regulation in an in vitro breast cancer or malignant melanoma setting will contribute to the search of mechanisms that can be exploited to therapeutically target HLA-G-mediated immune suppression in cancer. In the present study, we evaluated the extend of HLA class Ia and Ib expression in two breast cancer (MDA-MB-231, MCF-7) and two malignant melanoma (FM-55M2, FM-56) cell lines focusing primarily on HLA-G. We investigated the feasibility to manipulate expression of HLA-G by testing the effect of IFN-γ and 5-aza-dC on HLA-G expression in the cell lines. The results indicate that methylation-mediated repression of HLA-G can be reversed by treatment with 5-aza-dC and further induced by IFN-γ. However, HLA-G expression was not affected by IFN-γ alone. HLA-G expression is dominated by the presence of HLA-G1 followed by HLA-G2/4 and HLA-G3 isoforms. Furthermore, treatment with IFN-γ or 5-aza-dC generally promoted upregulation of most HLA class Ia molecules on the surface of the investigated cell lines, however, with some variability between the treatment conditions. Expression of HLA-E and HLA-DR was more heterogeneous between the cell lines.

## 2. Results

### 2.1. HLA Cell Surface Expression in Breast Cancer and Malignant Melanoma Cell Lines upon IFN-γ Stimulation

The role of IFN-γ in regulation of immunological mechanisms has been a great area of research. In the current study, the effect of IFN-γ on surface expression of HLA-A, HLA-B, HLA-C, HLA-E, HLA-G, and HLA-DR on MDA-MB-231, MCF-7, FM-55M2, and FM-56 cell lines was investigated by flow cytometry. Based on previously published studies, all cell lines were treated with 24–30 ng/mL IFN-γ and analyzed after two days of treatment [32]. Untreated JEG-3 cells, a choriocarcinoma cell line, was included as a positive control for HLA-G expression during visualization of the flow cytometry results. Results are presented as median fluorescence intensity (MFI) reflecting the expression level of each marker. For every stimulated sample, there was an untreated control sample and the effect of IFN-γ was analyzed as a direct comparison between these. It should be noted that MFI values cannot be directly compared between cell lines as analysis was performed as separate experiments.

**Figure 1:**
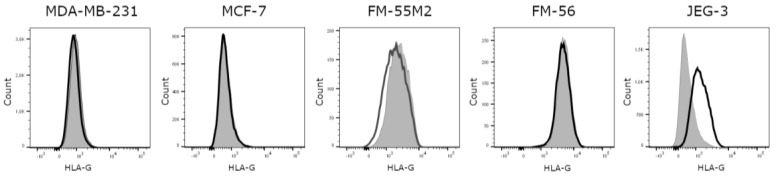
Human leukocyte antigen (HLA)-G surface expression on cancer cell lines. Representative histograms showing the expression of HLA-G on untreated cells compared to an isotype control. MDA-MB-231, MCF-7, FM-55M2, and FM-56 cell lines are HLA-G-negative, whereas JEG-3 cells are HLA-G-positive. Filled grey: Cells stained with isotype antibody as a negative control for HLA-G expression; solid black: Untreated cells stained with anti-HLA-G antibody.

Based on the comparison of untreated cell lines and an isotype control, it was evident that all investigated cell lines are HLA-G-negative, except for the HLA-G-positive JEG-3 control cell line (Figure 1). Representative flow cytometry data for all HLA molecules for all cell lines before and after treatment can be found in supplementary Figure S1 and S2. Unstimulated MDA-MB-231 cells expresses HLA-A and HLA-B, some HLA-C and HLA-E and no HLA-DR, whereas unstimulated MCF-7 cells expresses HLA-A, some HLA-E, and no HLA-B, HLA-C, or HLA-DR. The unstimulated FM-55M2 cell line expresses HLA-A and HLA-B, very low levels of HLA-E and HLA-DR, and no HLA-C, whereas the unstimulated FM-56 cell line expresses some HLA-B and HLA-DR and no HLA-A, HLA-C, or HLA-E. The JEG-3 cell line expresses HLA-C, HLA-E, some HLA-A and HLA-B, and no HLA-DR. The slightly negative MFI values observed in the results are caused by technicalities of flow cytometry making it possible to have a negative range of fluorescence intensity, when the background signal is automatically subtracted during acquisition of the data. All cases of negative values indicate no expression of the respective HLA molecule. Furthermore, the baseline level of the different HLA molecules can present with a relatively high MFI value even if there is no expression. This is caused by the adjustment of voltages of the flow cytometer in order to obtain the best separation of cell populations.

**Figure 2:**
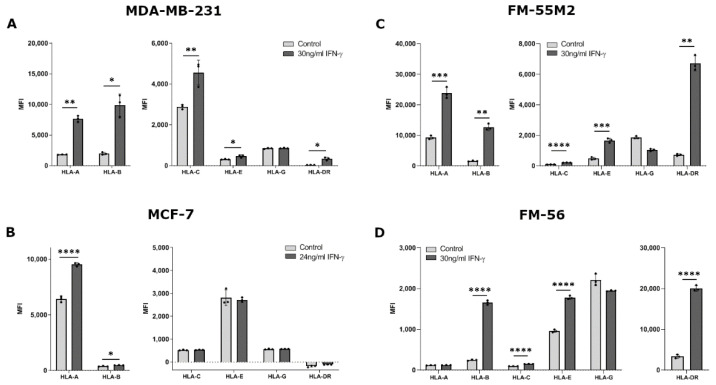
HLA surface expression on cancer cell lines stimulated with interferon (IFN)-γ. Flow cytometry analysis of HLA-A, HLA-B, HLA-C, HLA-G, HLA-E, and HLA-DR expression. (**A**) MDA-MB-231 cells treated with 30 ng/mL IFN-γ for two days. (**B**) MCF-7 cells treated with 24 ng/mL IFN-γ for two days. (**C**) FM-55M2 cells treated with 30 ng/mL IFN-γ for two days. (**D**) FM-56 cells treated with 30 ng/mL IFN-γ for two days. Shown are median fluorescence intensity (MFI) with mean ± SD, each dot represents one sample. All cell lines are HLA-G-negative and the shown MFI level of control samples represent the background. * *p* < 0.05, ** *p* < 0.01, *** *p* < 0.001, **** *p* < 0.0001 (Student’s unpaired *t*-test with Welch’s correction).

The ability of IFN-γ to change HLA surface expression was similar for most HLA molecules between all four treated cell lines. Treating MDA-MB-231 cells with IFN-γ for two days led to a significant increase in surface expression of HLA-A, HLA-B, HLA-C, HLA-E, and HLA-DR (HLA-A, *p* = 0.0024; HLA-B, *p* = 0.0165; HLA-C, *p* = 0.0093; HLA-E, *p* = 0.0200; HLA-DR, *p* = 0.0190) (Figure 2A). However, expression of HLA-G was not affected by IFN-γ. For MCF-7 cells, IFN-γ stimulated a significant upregulation of HLA-A and HLA-B, but did not affect the expression of the other HLA molecules (HLA- A, *p* < 0.0001; HLA-B, *p* = 0.0123) (Figure 2B). FM-55M2 cells treated with IFN-γ for two days had a significantly higher expression of all tested HLA molecules except for HLA-G expression (HLA-A, *p* = 0.0002; HLA-B, *p* = 0.0026; HLA-C, *p* < 0.0001; HLA-E, *p* = 0.0002; HLA-DR, *p* = 0.0020) (Figure 2C). Similarly, for FM-56 cells, IFN-γ stimulated a significant upregulation of all HLA molecules except HLA-A and HLA-G (HLA-B, *p* < 0.0001; HLA-C, *p* < 0.0001; HLA-E, *p* < 0.0001; HLA-DR, *p* < 0.0001) (Figure 2D). Additionally, malignant melanoma cell lines were treated with IFN-γ for four days to test the ability of IFN-γ to induce HLA-G expression after a longer incubation period. There was no increased effect of IFN-γ upon four days of culture compared to two days (Figure S3) and the long incubation period was therefore not tested with the remaining cell lines.

### 2.2. HLA Cell Surface Expression in Breast Cancer and Malignant Melanoma Cell Lines upon Demethylation Treatment

According to previous studies, epigenetic regulation such as DNA methylation plays a role in regulation of HLA expression. To further explore this, breast cancer and malignant melanoma cell lines were treated with the demethylating agent 5-aza-dC and surface expression of HLA-A, HLA-B, HLA-C, HLA-E, HLA-G, and HLA-DR was investigated by flow cytometry. Based on previously published studies, cells were treated with two different concentrations, 10 µM and 100 µM, of 5-aza-dC and analyzed on day three and six after treatment [33,35,36]. Representative flow cytometry data for all cell lines before and after treatment can be found in supplementary Figure S1 and S2.

It became evident during the analysis that treatment with 5-aza-dC promoted an increase in granularity of the cells causing an increase in autofluorescence for markers detected with fluorochromes with low emission wavelength. This was the case for HLA-C and HLA-G conjugated to PE and BV510 fluorochromes, respectively. The inclusion of isotype controls did not compensate for the increase in autofluorescence potentially causing a false increase in MFI for the treated samples. Furthermore, whether the potential effect of the change in autofluorescence was equal between isotypes and specific markers and between different samples was not transparent and isotype controls was therefore not included in the analysis. Consequently, the interpretation of HLA-C and HLA-G that are poorly expressed proteins must be done with caution.

**Figure 3:**
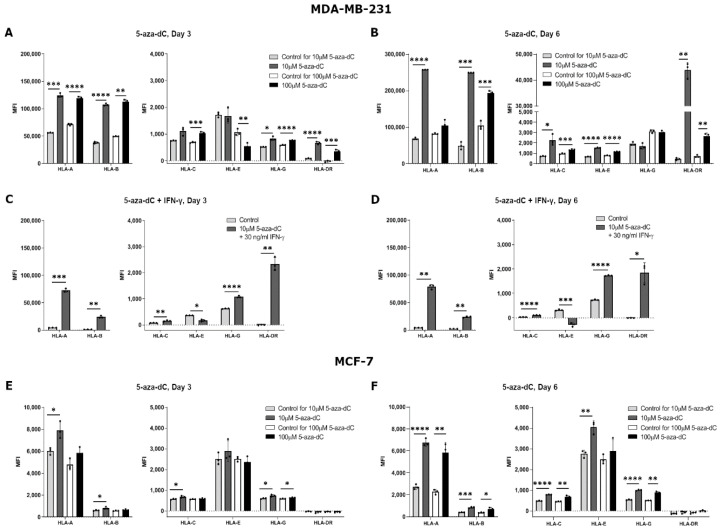
HLA surface expression on breast cancer cell lines stimulated with 5-aza-2′-deoxycytidine (5-aza-dC). Flow cytometry analysis of HLA-A, HLA-B, HLA-C, HLA-G, HLA-E, and HLA-DR expression. (**A**,**B**) MDA-MB-231 cells treated with either 10 µM or 100 µM 5-aza-dC for three (**A**) and six days (**B**). (**C**,**D**) MDA-MB-231 cells treated with 10 µM 5-aza-dC and 30 ng/mL IFN-γ for three (**C**) and six days (**D**). (**E**,**F**) MCF-7 cells treated with either 10 µM or 100 µM 5-aza-dC for three (**E**) and six days (**F**). Shown are median fluorescence intensity (MFI) with mean ± SD, each dot represents one sample. The shown MFI level of control samples represent a combination of background and baseline expression. * *p* < 0.05, ** *p* < 0.01, *** *p* < 0.001, **** *p* < 0.0001 (Student’s unpaired *t*-test with Welch’s correction).

The effect of 5-aza-dC on the HLA surface expression was highly variable both between the different HLAs and the different cell lines. Results for MDA-MB-231 cells treated with 10 µM 5-aza-dC for three days showed a significant upregulation in HLA-A, HLA-B, HLA-G, and HLA-DR surface expression, but no change for HLA-C and HLA-E (HLA-A, *p* = 0.0009; HLA-B, *p* < 0.0001; HLA-G, *p* = 0.0177; HLA-DR, *p* < 0.0001) (Figure 3A). When increasing the concentration to 100 µM 5-aza-dC, MDA-MB-231 cells had an increased expression of all HLA molecules after three days of treatment except for HLA-E expression, which seemed to decrease (HLA-A, *p* < 0.0001; HLA-B, *p* = 0.0013; HLA-C, *p* = 0.0007; HLA-E, *p* = 0.0069; HLA-G, *p* < 0.0001; HLA-DR, *p* = 0.0006) (Figure 3A). Treating the cells with 10 µM 5-aza-dC for six days increased the expression of all HLA molecules except for HLA-G (HLA-A, *p* < 0.0001; HLA-B, *p* = 0.0009; HLA-C, *p* = 0.0353; HLA-E, *p* < 0.0001; HLA-DR, *p* = 0.0016) (Figure 3B). Furthermore, after six days of treatment with 100 µM 5-aza-dC, surface expression of all HLA molecules increased except for HLA-A and HLA-G expression, which did not change (HLA-B, *p* = 0.0003; HLA-C, *p* = 0.0010; HLA-E, *p* < 0.0001; HLA-DR, *p* = 0.0043) (Figure 3B). For MCF-7 cells, we observed slightly different results. Three days of treatment with 10 µM 5-aza-dC led to a significant upregulation of HLA-A, HLA-B, HLA-C, and HLA-G and no change for HLA-E and HLA-DR expression (HLA-A, *p* = 0.0174; HLA-B, *p* = 0.0269; HLA-C, *p* = 0.0235; HLA-G, *p* = 0.0123) (Figure 3E). When increasing the concentration to 100 µM 5-aza-dC, there was only a small increase in HLA-G expression on MCF-7 cells upon three days of treatment (HLA-G, *p* = 0.0317) (Figure 3E). After six days of treatment with 10 µM 5-aza-dC, surface expression increased for all HLA molecules, now also for HLA-E, but still with no change in HLA-DR expression (HLA-A, *p* < 0.0001; HLA-B, *p* = 0.0004; HLA-C, *p* < 0.0001; HLA-E, *p* = 0.0033; HLA-G, *p* < 0.0001) (Figure 3F). Furthermore, after six days with 100 µM 5-aza-dC, results were similar to the other treatments with an increase in HLA-A, HLA-B, HLA-C, and HLA-G surface expression, but with no change for HLA-E and HLA-DR expression (HLA-A, *p* = 0.0028; HLA-B, *p* = 0.0198; HLA-C, *p* = 0.0026; HLA-G, *p* = 0.0065) (Figure 3F).

**Figure 4:**
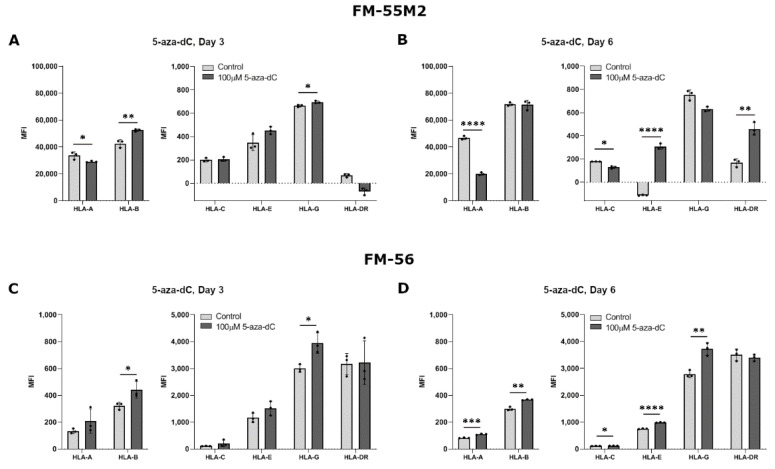
HLA surface expression on malignant melanoma cell lines stimulated with 5-aza-dC. Flow cytometry analysis of HLA-A, HLA-B, HLA-C, HLA-G, HLA-E, and HLA-DR expression. (**A**,**B**) FM-55M2 cells treated with 10 µM 5-aza-dC for three (**A**) and six days (**B**). (**C**,**D**) FM-56 cells treated with 10 µM 5-aza-dC for three (**C**) and six days (**D**). Shown are median fluorescence intensity (MFI) with mean ± SD, each dot represents one sample. The shown MFI level of control samples represent a combination of background and baseline expression. * *p* < 0.05, ** *p* < 0.01, *** *p* < 0.001, **** *p* < 0.0001 (Student’s unpaired *t*-test with Welch’s correction).

As we mostly observed the biggest change in HLA expression upon stimulation with 100 µM compared to 10 µM 5-aza-dC in the breast cancer cell lines, stimulation of the malignant melanoma cell lines was performed with only 100 µM. Treatment of FM-55M2 with 100 µM 5-aza-dC for three days promoted a significant upregulation of HLA-B and HLA-G expression, whereas there was a decrease in HLA-A expression and no change in HLA-C, HLA-E, and HLA-DR expression (HLA-A, *p* = 0.0476; HLA-B, *p* = 0.0046; HLA-G, *p* = 0.0180) (Figure 4A). After six days of treatment, FM-55M2 cells had a lower expression of HLA-A and HLA-C, a higher expression of HLA-E and HLA-DR, and no change in HLA-B and HLA-G expression (HLA-A, *p* < 0.0001; HLA-C, *p* = 0.0131; HLA-E, *p* < 0.0001; HLA-DR, *p* = 0.0014) (Figure 4B). For FM-56 cells, treatment with 5-aza-dC for three days led to a significant increase in surface expression of HLA-B and HLA-G and a tendency towards higher expression of most of the other HLA molecules (HLA-B, *p* = 0.0383; HLA-G, *p* = 0.0166) (Figure 4C). However, the cells displayed a significantly higher expression of all HLA molecules after six days of treatment except for HLA-DR (HLA-A, *p* = 0.0009; HLA-B, *p* = 0.0011; HLA-C, *p* = 0.0392; HLA-E, *p* < 0.0001; HLA-G, *p* = 0.0039) (Figure 4D).

Finally, we investigated whether a simultaneous treatment with both 5-aza-dC and IFN-γ had an increased effect on HLA surface expression. MDA-MB-231 cells were used as a representative cell line and treated with 10 µM 5-aza-dC and 30 ng/mL IFN-γ for three and six days. The cells displayed a significant upregulation of expression of all HLA molecules both after three and six days of treatment except for HLA-E expression, which decreased (HLA-A, *p* = 0.0007, *p* = 0.0011; HLA-B, *p* = 0.0025, *p* = 0.0016; HLA-C, *p* = 0.0025, *p* < 0.0001; HLA-E, *p* = 0.0129, *p* = 0.0002; HLA-G, *p* < 0.0001, *p* < 0.0001; HLA-DR, *p* = 0.0038, *p* = 0.0163; day three and six, respectively) (Figure 3C,D). In contrast to treating the cells with either IFN-γ or 5-aza-dC for six days, the combined treatment led to a marked increase in HLA-G both after three and six days of treatment.

### 2.3. HLA-G mRNA Expression in Breast Cancer and Malignant Melanoma Cell Lines

**Figure 5:**
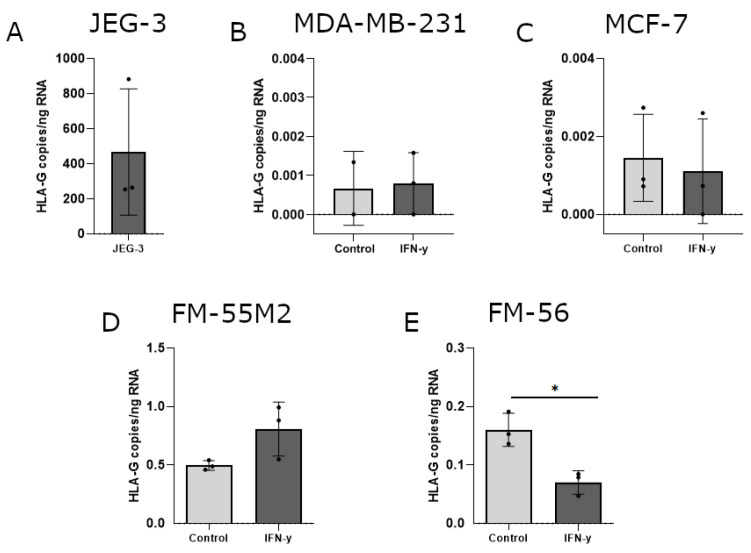
HLA-G mRNA expression in cancer cell lines stimulated with interferon (IFN)-γ. The effect of IFN-γ on the transcriptional level of HLA-G was determined by digital droplet (dd)PCR and presented as number of HLA-G copies per amount of RNA. (**A**) JEG-3 control cells with high HLA-G expression. (**B**) MDA-MB-231 cells treated with 30 ng/mL IFN-γ for two days. (**C**) MCF-7 cells treated with 24 ng/mL IFN-γ for two days. (**D**) FM-55M2 cells treated with 30 ng/mL IFN-γ for two days. (**E**) FM-56 cells treated with 30 ng/mL IFN-γ for two days. Shown are mean ± SD, each dot represents one sample. * *p* < 0.05 (Student’s unpaired t-test with Welch’s correction).

The ability of IFN-γ and 5-aza-dC to induce HLA-G surface expression was variable between the different cell lines and to elaborate on the uncertainty about these results, induction of HLA-G was investigated on mRNA level. Furthermore, several previous studies on HLA-G regulation focus on mRNA expression including investigation of the soluble HLA-G isoforms. The level of HLA-G mRNA was calculated as HLA-G copies per amount of input RNA initially used for cDNA synthesis and subsequent digital droplet (dd)PCR. The HLA-G-positive choriocarcinoma cell line JEG-3 was included as a control (Figure 5A). As expected, all untreated cells had no or very low levels of HLA-G mRNA. For both breast cancer cell lines, IFN-γ treatment did not have an effect on HLA-G mRNA levels (Figure 5B,C). The effect of IFN-γ on HLA-G mRNA expression in malignant melanoma cell lines displayed a marked heterogeneity. The level of HLA-G increased slightly in FM-55M2 cells upon two days of culture with IFN-γ (Figure 5D), but IFN-γ seemed to lower the HLA-G level in FM-56 cells after two days of culture (*p* = 0.0109) (Figure 5E). However, HLA-G levels were particularly low in FM-55M2 and FM-56 cells upon treatment with IFN-γ questioning the effect of IFN-γ on the transcriptional level of HLA-G.

**Figure 6:**
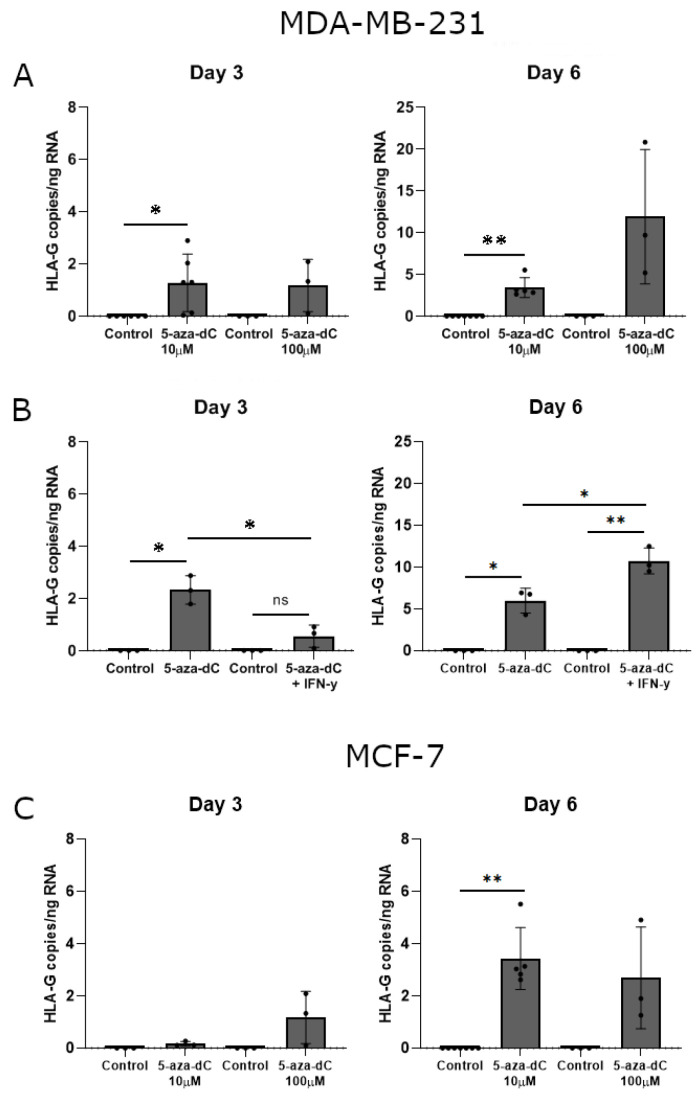
HLA-G mRNA expression in stimulated breast cancer cell lines. The effect of 5-aza-dC on the transcriptional level of HLA-G was determined by ddPCR and presented as number of HLA-G copies per amount of RNA. (**A**) MDA-MB-231 cells treated with 10 μM or 100 μM 5-aza-dC for three and six days. (**B**) MDA-MB-231 cells treated with a combination of 10 μM 5-aza-dC and 30 ng/mL IFN-γ for three and six days. (**C**) MCF-7 cells treated with 10 μM or 100 μM 5-aza-dC for three and six days. Shown are mean ± SD, each dot represents one sample. * *p* < 0.05, ** *p* < 0.01 (Student’s unpaired *t*-test with Welch’s correction).

Interestingly, treatment with 10 μM 5-aza-dC promoted a significant upregulation of HLA-G mRNA expression in MDA-MB-231 cells after both three and six days of culture (Day 3, *p* = 0.0360; Day 6, *p* = 0.0029) (Figure 6A). Treatment with 100 μM 5-aza-dC also seemed to induce HLA-G mRNA expression, though not significantly (Figure 6A). Notably, HLA-G levels were higher on day six of treatment compared to day three for corresponding samples. For MCF-7 cells, only 10 μM 5-aza-dC induced a significant upregulation of HLA-G mRNA upon six days of culture (Day 6, *p* = 0.0029) (Figure 6C). Treatment with 100 μM 5-aza-dC seemed also to increase HLA-G levels; however, there was a variation between samples (Figure 6C).

**Figure 7:**
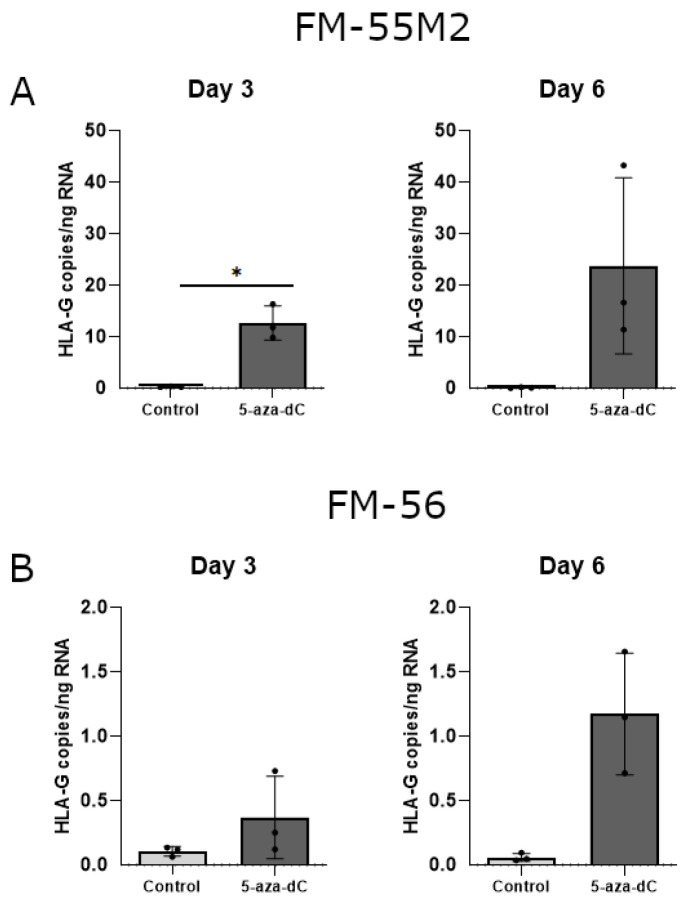
HLA-G mRNA expression in stimulated malignant melanoma cell lines. The effect of 5-aza-dC on the transcriptional level of HLA-G was determined by ddPCR and presented as number of HLA-G copies per amount of RNA. (**A**) FM-55M2 cells treated with 100 μM 5-aza-dC for three and six days. (**B**) FM-56 cells treated with 100 μM 5-aza-dC for three and six days. Shown are mean ± SD, each dot represents one sample. * *p* < 0.05 (Student’s unpaired *t*-test with Welch’s correction).

With some variations in the data, there was a strong tendency towards induction of HLA-G mRNA upon treatment with 5-aza-dC in both malignant melanoma cell lines (Figure 7A,B). Treatment of FM-55M2 cells with 100 μM 5-aza-dC for three days promoted a significant induction of HLA-G (*p* = 0.0234) (Figure 7A). Furthermore, the level of HLA-G was much higher in FM-55M2 cells compared to FM-56 cells.

Finally, we again investigated the additive effect of simultaneous treatment with 5-aza-dC and IFN-γ on the mRNA expression in MDA-MB-231 cells. Upon three days of treatment, the combined effect of 5-aza-dC and IFN-γ had no significant effect on HLA-G expression compared to the control and did not reach the level of HLA-G upon 5-aza-dC treatment alone (5-aza-dC, *p* = 0.0178; 5-aza-dC vs. 5-aza-dC + IFN-γ, *p* = 0.0112) (Figure 6B). On day six there was a significant increase in HLA-G expression both compared to the control and to 5-aza-dC treatment alone in agreement with the increase in surface expression observed by flow cytometry (5-aza-dC, *p* = 0.0196; 5-aza-dC + IFN-γ, *p* = 0.0068; 5-aza-dC vs. 5-aza-dC + IFN-γ, *p* = 0.0182) (Figure 6B).

### 2.4. Verification of HLA-G Demethylation

**Figure 8:**
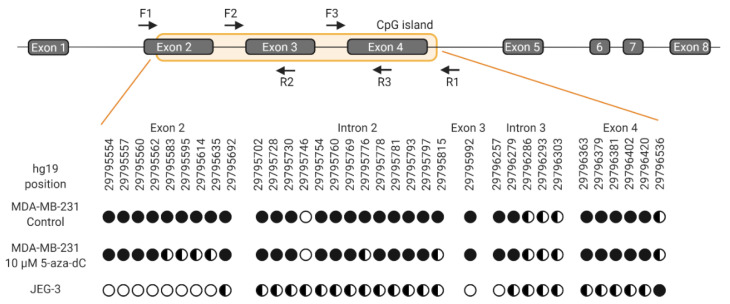
Schematic representation of the *HLA-G* gene and methylation status of selected CG sites. For bisulfite sequencing, three overlapping amplicons covering the CpG island were used. Forward and reverse primers indicated by arrows are matched in pairs F1R2, F2R3, and F3R1, respectively. *HLA-G* nomenclature follows NCBI annotation and prediction of CpG island is from USCS Genome Browser. Black circles indicate methylation, empty circles indicate no methylation and half-filled circles indicate partly methylation. Multiple CG dinucleotides in the CpG island could not be determined due to polymerase slippage in T rich areas. Therefore, an analysis of all CG sites within the CpG island was not possible.

Demethylation of the *HLA-G* gene upon treatment with 5-aza-dC was verified by bisulfite sequencing. A comparison of selected CG sites within the CpG island covering exon 2-4 of the *HLA-G* gene was made between MDA-MB-231 cells and JEG-3 cells (Figure 8). Untreated MDA-MB-231 cells were methylated on investigated sites, whereas cells treated with 5-aza-dC displayed a pattern of partly methylation on a number of CG sites corresponding to the pattern observed in HLA-G-positive JEG-3 cells (Figure 8). The number of unmethylated CG sites was lower in treated MDA-MB-231 cells compared with JEG-3 cells, which could represent the difference in surface expression between the two cell lines. Hence, these results indicate that DNA demethylation was associated with the slight but significant increase in HLA-G expression after 5-aza-dC treatment.

### 2.5. Detection of HLA-G mRNA Isoforms in Breast Cancer and Malignant Melanoma Cell Lines upon 5-aza-dC Treatment

As described previously, there are seven verified HLA-G isoforms. The different isoforms might constitute different functional forms of HLA-G, also emphasized by a marked heterogeneity in isoform expression observed within the same tumor for some cancers [22]. To further investigate the expression of HLA-G upon 5-aza-dC treatment, we assessed the HLA-G mRNA isoform profile. We performed fragment analysis by capillary electrophoresis of reverse transcriptase PCR products from MDA-MB-231, MCF-7, FM-56, and FM-55M2 cells treated with 5-aza-dC. For the malignant melanoma cell lines, we also performed the analysis on unstimulated control cells, since ddPCR showed low levels of HLA-G mRNA before treatment.

The presence of the 14 bp 3′ UTR insertion polymorphism can facilitate further splicing of 92 bp from the 3′UTR from all seven HLA-G isoforms, thus affecting analysis of the isoform landscape. In order to detect specific splice variants of the isoforms in the fragment analysis, the presence of the 14 bp polymorphism in all cell lines was investigated by TaqMan assay (Table 1). Breast cancer cell lines MDA-MB-231 and MCF-7 are both homozygous for the 14 bp deletion. The FM-56 and JEG-3 cell lines are homozygous for the 14 bp insertion while the FM-55M2 cell line are heterozygous with respect to the 14 bp sequence polymorphism.

**Figure 9:**
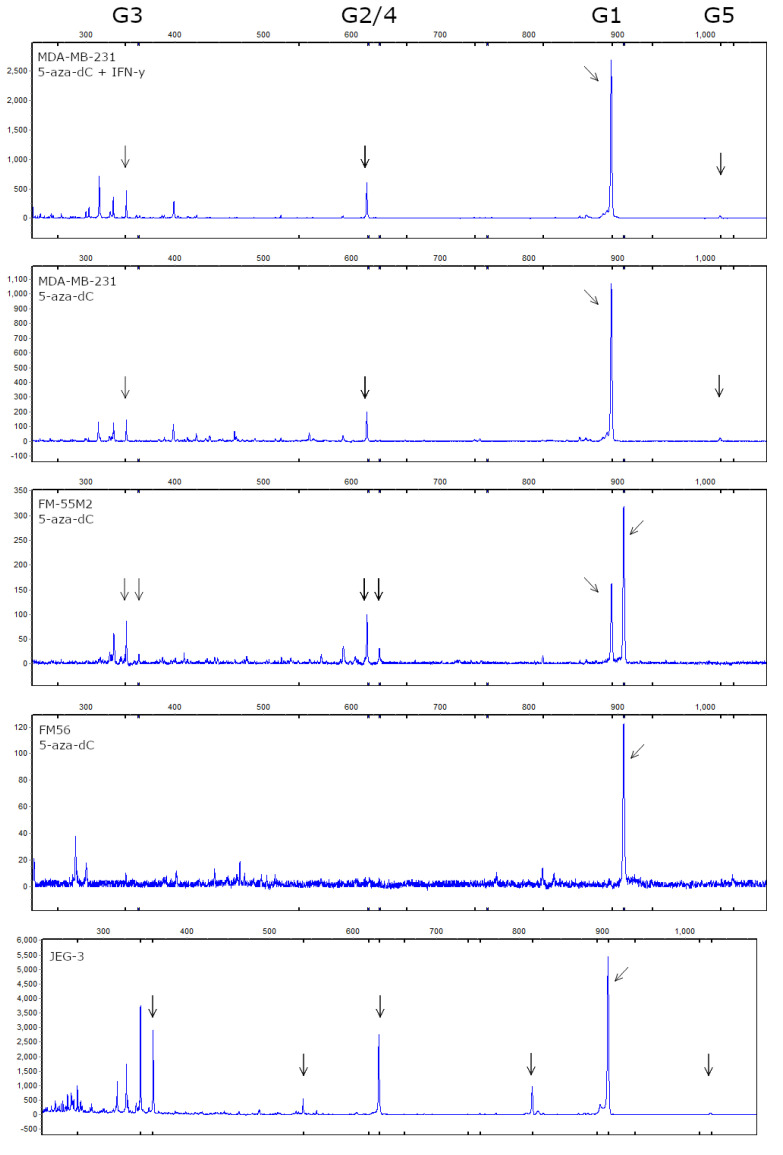
HLA-G isoform profile of stimulated breast cancer and malignant melanoma cell lines. Determination of HLA-G mRNA isoform expression was performed by fragment analysis. From the top is shown representative electropherograms from MDA-MB-231 cells treated with both 5-aza-dC and IFN-γ, and MDA-MB-231, FM-55M2 and FM-56 cells treated with 5-aza-dC, respectively. An electropherogram from the HLA-G-positive JEG-3 cell line is shown in the bottom as a positive control. Arrows highlight detected peaks corresponding to HLA-G1, -G2/4, -G3, and -G5 as listed on top. For FM-55M2 cells two peaks for each isoform indicate the heterozygous 14 bp ins/del phenotype with the additional +14 bp isoform. For JEG-3 cells peaks corresponding to the 14 bp insertion and the Δ92 bp splice variant is detected.

As described, the 14 bp polymorphism results in isoforms of different lengths depending on splicing of the 92 bp but for simplicity, we refer to the isoforms as G1-G7 for all cell lines. We were able to identify peaks in the electropherograms corresponding to the predicted sizes of HLA-G1 (894 bp), G2/4 (619 bp), -G3 (345 bp), and -G5 (1017 bp). Representative electropherograms are shown in Figure 9, as well as an electropherogram based on mRNA from the JEG-3 cell line that expresses high levels of HLA-G. In the MDA-MB-231 and FM-55M2 cells, we detected the highest signal from HLA-G1 followed by HLA-G2/4 and HLA-G3. In the samples with the highest signal, we were also able to detect a peak corresponding to HLA-G5. For FM-55M2 cells, we detected a peak corresponding to HLA-G1 both with and without the 14 bp insertion as well as small peaks for HLA-G2/4 and -G3 with the 14 bp insertion. In FM-56 cells, we detected only HLA-G1. For MCF-7 cells and non-treated FM-55M2 and FM56 cells, we failed to detect any peaks due to low signal intensity. For the HLA-G-positive cell line JEG-3, we confirmed the presence of HLA-G1-5 isoforms (Figure 9). As a result of the presence of the 14 bp insertion, we also detected a Δ92 bp splice variant for HLA-G1 and HLA-G2/4.

The signal intensity was generally low for breast cancer and malignant melanoma cell lines compared to JEG-3 cells and thus expression levels of the low-expressed HLA-G isoforms presumably fell below the limit of detection preventing a semi-quantitative analysis of the distribution of HLA-G isoforms in the samples. The quality of the original RNA purified from treated cells was checked by automated electrophoresis and was consistently high for all samples, ruling out that poor RNA quality was the cause of missing signals in the fragment analysis (data not shown).

## 3. Discussion

HLA-G is a well-studied molecule and information regarding its regulatory role in cancer is accumulating. To explore how cancer cell lines regulate HLA expression and whether HLA-G expression can be induced by the cytokine IFN-γ and the demethylating agent 5-aza-dC, we treated selected malignant melanoma and breast cancer cell lines with these two compounds, both individually and combined, and measured the effect on mRNA and cell surface expression levels. Expression of HLA-A, HLA-B, HLA-C, and HLA-E were included in this study in order to broaden the knowledge on how IFN-γ and 5-aza-dC affect both classical and non-classical HLA molecules giving that a concomitant deregulation of HLA class Ia and Ib molecules is associated with immune evasion in cancer [37]. Finally, the HLA class II molecule HLA-DR was included. HLA-DR is less studied with respect to cancer but increasing evidence suggests that HLA-DR might have a predictive role for malignant melanoma. The choice of stimulating cancer cell lines with both IFN-γ and 5-aza-dC addresses two different molecular mechanisms for regulation of *HLA* gene expression. A summary of the findings can be found in Table 2.

In the present study, IFN-γ treatment had no effect on HLA-G mRNA level or protein expression in any of the cell lines. The HLA-G mRNA seemed to decrease in FM-56 cells, but a decrease in HLA-G might be attributed to the uncertainty in the noticeably low mRNA level detected with ddPCR analysis. With some variability between cell lines, IFN-γ mainly promoted an upregulation of the other investigated HLA molecules. The ability of IFN-γ to induce HLA class Ia antigen expression in cancer cells has been observed previously. An early study by Jabrane-Ferrat et al. detected HLA class Ia and HLA-DR surface expression on MDA-MB-231 and MCF-7 breast cancer cell lines that were increased by IFN-γ treatment [38]. In the present study, a similar increase was observed for only MDA-MB-231 cells. A study by Rodríguez et al. found that out of 57 cell lines, only two (ESTDAB-004 and ESTDAB-159) did not increase mRNA or surface expression of HLA-ABC upon treatment with IFN-γ [39]. The absence of IFN-γ-mediated expression of HLA-ABC in one of the cell lines was presumably due to epigenetic blocking of the IFN-γ response element IRF-1, since expression could be induced by a concomitant treatment with both IFN-γ and the demethylation agent 5-aza-dC.

When it comes to HLA-G surface expression, HLA-G expression is rarely detected on in vitro established cancer cell lines making it challenging to study the molecular function of HLA-G. A comprehensive study by Polakova and Russ on 75 different cancer cell lines including melanoma and breast cancer (e.g., MDA-MB-231 and MCF-7) imply that under normal culture conditions the cell surface expression of HLA-G is absent in most cancer cell lines across different origin [40]. Similarly, a study by Frumento et al. of 45 melanoma cell lines showed no evidence of neither mRNA or protein HLA-G expression, nor that it could be induced by IFN-γ treatment [41]. The four investigated cell lines in the present study is also HLA-G-negative. Surprisingly, there is a discrepancy between the frequent expression of HLA-G in tumor lesions and the lack of HLA-G expression in most cancer cell lines. This reflect a lack of selection pressure for HLA-G expression for cells that are cultured in vitro. This is supported by a study by Rouas-Freiss et al. showing that the Fon melanoma cell line loses cell-surface expression of HLA-G1 upon long-term culture [42], which is also seen in previous studies on renal carcinoma cell lines [43] and ovarian carcinoma cell lines [44]. Furthermore, Rouas-Freiss et al. showed that treatment with IFN-γ and 5-aza-dC demethylating agent increased HLA-G1 cell-surface expression on HLA-G-positive cells. However, HLA-G1 re-expression was not induced in HLA-G-negative cells upon treatment. The concentration of IFN-γ in patients are often lower in both peripheral blood and in the tumor microenvironment than the concentration used in the present study that was chosen with the aim to boost HLA-G expression [45,46]. As we do not observe an effect of IFN-γ on the expression of HLA-G it is unlikely that IFN-γ could be an independent inducer of HLA-G in the tumor microenvironment at lower concentrations.

Whereas we did not see an upregulation of HLA-G upon IFN-γ stimulation, we did see a clear upregulation of HLA-G upon treatment with a combination of IFN-γ and 5-aza-dC. These results indicate that IFN-γ might be involved in maintaining HLA-G expression rather than inducing expression. This is also observed in one of our previous studies with a focus on reproductive immunology [32].

The analysis of HLA-DR expression upon IFN-γ treatment aids to the emerging interest in the role of HLA-DR during cancer immune surveillance. When stimulating the melanoma cell lines with IFN-γ, expression of HLA-DR significantly increased, which was consistent with observations of other studies [47,48]. Although expression of HLA class II molecules is usually restricted to antigen-presenting cells, studies show that almost 50% of melanomas constitutively express HLA class II, and that HLA-DR expression in malignant melanoma correlates with increased motility, migration, and expression of adhesion molecules [47]. Thus, expression of HLA-DR in malignant melanoma correlates with a worse prognosis. On the other hand, HLA-DR expression is associated with therapeutic response in a cohort of anti-PD1 treated patients suggesting that HLA-DR could be a possible positive predictor of anti PD-1 immunotherapy [48].

Lack of HLA-G expression seems to be independent of repression of other HLA class I and II molecules as expression of these is observed even when HLA-G is absent. The difference in expression of HLA class Ia molecules and HLA-G might be attributed to differences in the regulatory elements upstream from the translation start site of each *HLA* gene. Although many variable sites in the promoter region of *HLA* genes are conserved, variability in the transcription factor target sites most likely influences the differential regulation. The IFN-stimulated response element (ISRE) located in the proximal promoter region of *HLA class I* genes displays locus-specific nucleotide variation between the different *HLAs* leading to differences in IFN-γ-induced expression levels of *HLA class I* genes [49]. The ISRE sequence of the *HLA-G* gene is the most divergent compared to the ISRE consensus sequence preventing binding of interferon regulatory factors and raising the issue of whether the *HLA-G* ISRE is fully functional [50]. The *HLA-G* gene also contains other regulatory elements, which are not functional (reviewed in [50]). Combined with ISRE is the Enhancer A (EnhA) module and next to it a SXY module. The NFĸB responsive element of EnhA is more efficient for *HLA class Ia* than for *class Ib* genes [51]. The SXY module is crucial for CIITA; however, divergence of the X2 and Y elements prevents CIITA transactivation of *HLA-G* [51,52]. Most likely, several mechanisms play a role in regulation of HLA protein expression in different cases of cancer. Differences in IFN-γ-mediated inducibility of HLA molecules between cancer cell lines may reflect differences in the cytokine reactivity and nature of the original tumor cells. Understanding the mechanisms by which tumors react to IFN-γ signaling will contribute to therapeutic decision making and help predicting response to immunotherapy, as it might represent a potential mechanism of resistance to therapy. In relation to this, the differential regulation of HLA class I molecules must be taken into consideration and might show as an advantage when choosing a treatment strategy.

The first evidence that DNA hypermethylation is used by cancer cells to switch off *HLA class I* genes was provided by Serrano et al. in 2001, where treatment with 10 µM 5-aza-dC for 15 days restored HLA-ABC expression on the otherwise HLA-ABC-negative melanoma cell line MSR3-mel [53]. Upon demethylation, HLA-ABC expression could be further induced by IFN-γ treatment. The ability to induce HLA-ABC molecules by exposure to 5-aza-dC was furthermore seen for the melanoma cell line Mel 275, also leading to induced recognition by cytotoxic T lymphocytes [54]. In the present study, we also observed a significant upregulation of HLA class Ia molecules after treatment with 5-aza-dC on all cell lines that normally expressed HLA class Ia. For HLA-E, the results were more diverse. Expression of HLA-DR was not affected by 5-aza-dC treatment in MCF-7 and FM-56 cells, but was upregulated upon all treatment conditions in MDA-MB-231 cells and after six days of treatment in FM-55M2 cells. Finally, there was a tendency towards higher expression of HLA-G in all cell lines keeping in mind the uncertainty with the cell surface expression of HLA-G measured by flow cytometry. For future studies, an alternative analysis methodology that overcome the prerequisite of flow cytometry to analyze only cells with same size and granularity is recommended to fully elaborate on the ability of 5-aza-dC to induce HLA-G surface expression. Nevertheless, the verification of DNA demethylation by bisulfite sequencing and the demonstrated increase in HLA-G mRNA upon treatment indicated that *HLA-G* transcription is partly regulated by DNA methylation. Previous studies have also revealed that *HLA-G* gene transcription is inhibited by DNA methylation. A previous study has proven an induction of HLA-G in MCF-7 cells upon treatment with 5-aza-dC and IFN-γ [55]. However, this study was unable to detect cell surface expression of HLA-G by flow cytometry. Another study by Moreau et al. showed that treatment with 1, 10, and 100 µM 5-aza-dC for three days induced expression of HLA-G in selected cancer cell lines from embryonal carcinoma, leukemia, lymphomas, and melanoma [33]. Bisulfite sequencing of the 450 bp promoter region upstream from the transcription start site of *HLA-G* in some of the same cell lines showed that the degree of CpG methylation correlated with expression [56]. Similar results have been observed for the melanoma cell line OCM-1A where bisulfite sequencing revealed methylation of 87% of CpG sites in the promoter region of *HLA-G* before treatment compared to only 2% after treatment [57]. Furthermore, the level of methylation in the *HLA-G* promoter region seem to differ between normal ovarian surface epithelium and ovarian tumors, though not associated with the expression level [58]. Interestingly, this study showed that CpG sites related to a hypoxia response element (HRE) remained methylated even upon 5-aza-dC treatment of an ovarian cancer cell line suggesting a strong selection against loss of methylation in this region. Investigation of the *HLA-G* promoter region and part of the CpG island in colorectal cancer cell lines and choriocarcinoma cell lines have further showed a discrepancy between methylation level and HLA-G expression [59,60]. Similar results is observed in a database search on invasive breast carcinoma using the Wanderer tool, which visualizes gene expression and DNA methylation profiles obtained from the TCGA research network [61]. Breast tumor tissue seem to display a higher percentage of methylation at selected CG sites in the *HLA-G* gene compared with normal breast tissue; however, there is no significant difference in HLA-G expression. Combined, these findings suggest that DNA methylation is not the sole mechanism regulating HLA-G expression, especially in human tumors, where heterogeneous HLA-G expression can represent the complex interaction between cancer cells and factors in the tumor microenvironment that are not present in vitro.

Fragment analysis of cells treated with 5-aza-dC revealed that the predominant HLA-G isoform was HLA-G1, followed by HLA-G2/4 and HLA-G3. The HLA-G5 isoform was also observed for the MDA-MB-231 cells. However, there was a low signal intensity in the analysis possibly hiding the presence of other isoforms. This is emphasized by the high signal and clear presence of HLA-G1-5 in the HLA-G-positive JEG-3 cell line. The low signal intensity was presumably due to low mRNA levels in general, in spite of 5-aza-dC-mediated induction of low levels of HLA-G in otherwise HLA-G-negative cell lines. Furthermore, the discrepancy between the HLA-G mRNA levels detected by ddPCR and the low signal obtained by fragment analysis indicate that the latter technique is limited by assay sensitivity. To fully elucidate on the HLA-G isoform profile in the cancer cell lines upon 5-aza-dC treatment a more sensitive method such as RNA sequencing is required. When comparing the isoform profile and the protein level detected by flow cytometry, there is a clear correlation between the results. The HLA-G antibody used detects HLA-G1 and HLA-G5 isoforms and with all cell lines predominantly expressing HLA-G1, the surface expression level reflects the majority of the total HLA-G expression level. However, the surface expression of HLA-G is presumably higher than detected due to expression of HLA-G2-4 isoforms, which are not detected by the antibody used. The level of surface expression might especially be higher for JEG-3 cells that present with a relatively higher level of HLA-G1-5 isoforms compared to the other cell lines.

Altogether, our results support that reversal of methylation-mediated repression of HLA-G is a co-acting mechanism by which tumor cells can take advantage of in order to avoid immune recognition. Demethylation is a common mechanism exploited by cancer cells to induce gene transcription and can explain the difference between HLA-G expression in vivo and in vitro when there is no selection pressure from the tumor microenvironment. A similar model has been proposed before for melanoma cells. Expression of HLA-G in melanocytes can be induced during malignant transformation mediated by stress and epigenetic mechanisms otherwise employed by the cancer cells to facilitate the oncogenic transformation. However, adaptation to in vitro culture conditions might lead to methylation due to the lack of stress signals from the tumor microenvironment [62]. Other possible factors involved in signaling within the tumor microenvironment for breast cancer include the hormone receptor status. There seem to be an association between estrogen receptor (ER) status and HLA expression. The frequency of HLA class II-positive tumor cells are lower in ER positive breast carcinomas compared to ER-negative tumors [63]. Furthermore, ER signaling differentially modulates IFN-γ-mediated HLA class II induction in breast cancer cell lines as estradiol attenuates HLA-DR expression in ER-positive cells [64]. Interestingly, this correlates with the expression of HLA-DR observed in the present study. MDA-MB-231 cells are estrogen receptor-negative and display a marked induction in HLA-DR surface expression upon treatment. On the other hand, MCF-7 cells are estrogen receptor-positive with no expression of HLA-DR before or after treatment. Other cytokines, such as IL-4 also seems to be related to the hormone receptor status and to clinical outcome [65]. The extensive cytokine profile in the tumor microenvironment plays an important role in cancer progression. A previous study on MDA-MB-231 and MCF-7 cell lines show that IL-6 and IL-10 are associated with good prognosis in breast cancer [66]. Furthermore, distinct patterns of cytokines, including expression of IFN-γ, IL-4, and IL-10, have been observed in specific breast cancer groups compared to a healthy population [67]. Whether these cytokines might be involved in epigenetic changes such as DNA methylation is an interesting question to address in future studies.

It could be speculated that the HLA expression profile is reflected by the nature of the original cancer giving rise to the cancer cell lines. Both breast cancer cell lines originate from pleural effusion and display similar expression profiles for most HLA molecules except HLA-DR. Treatment with IFN-γ and 5-aza-dC seems to be more potent inducers of HLA surface expression on MDA-MB-231 cells compared to the MCF-7 cells. Whereas the FM-55M2 cells originate from lymph node metastasis, FM-56 cells originate from a primary tumor. Despite the nature of the cancer, the HLA expression profile are similar for the two cell lines except for HLA-A expression which seems to be low or absent on unstimulated FM-56 cells. Furthermore, the effect of IFN-γ and 5-aza-dC on HLA surface expression is similar for most HLAs on both cell lines. The HLA-G isoform profile is also similar for all four cell lines predominantly expressing HLA-G1. Consequently, there is no indication that the nature of the tumor is related to the expression of HLA molecules in the in vitro setting investigated in the present study.

A broader understanding of the mechanism by which HLA-G expression is regulated is essential in the development of immune checkpoint inhibitors targeted at HLA-G. The expression of HLA-G in tumor lesions should also be taken into consideration in the design of therapeutic strategies. Assuming that HLA-G is associated with poor clinical outcome the aim of therapeutic interventions should be to prevent a possible induction of its expression in tumors negative for HLA-G. The present study demonstrate two different approaches to manipulate HLA expression in an in vitro breast cancer and malignant melanoma setting and support a role for DNA methylation in regulation of HLA-G expression. The results presented here can pave the way for further research related to methylation-mediated regulation of HLA class I and class II expression. Furthermore, future studies investigating combinations of different cytokines present in the tumor microenvironment will be essential for the understanding of regulatory factors promoting epigenetic changes that affect HLA expression in primary tumors.

## 4. Materials and Methods

### 4.1. Cells and Culture Conditions

The current study was performed on four different cell lines from two different cancers: Human breast cancer cell lines MDA-MB-231 (pleural effusion) and MCF-7 (pleural effusion), and human malignant melanoma cell lines FM-56 (primary melanoma) and FM-55M2 (lymph node metastasis), which was kindly provided by Prof. Mads Hald Andersen, Herlev Hospital, Denmark. Human choriocarcinoma cell line JEG-3 (ATCC, Manassas, VA, USA, #HTB-36) was used as a HLA-G-positive control cell line. JEG-3 and MCF-7 cells were cultured in Eagle’s Minimum Essential Medium (EMEM, ATCC^®^-30-2003^TM^) and MDA-MB-231, FM-56, and FM-55M2 cells were cultured in Roswell Park Memorial Institute 1640 medium (RPMI, ThermoFisher, Waltham, MA, USA, #61870-010). Culture medium was supplemented with 10% heat-inactivated Fetal Bovine Serum (FBS; Sigma-Aldrich, St. Louis, MO, USA, #F9665). All cells were cultured at 37 °C and 5% CO_2_ and sub-cultured when they reached 75–80% confluency. For cell passaging and cell harvest, the cells were gently washed in phosphate-buffered saline (PBS, ThermoFisher, Waltham, MA, USA, #14190250) and trypsinized using 0.25% EDTA-Trypsin (ThermoFisher, Waltham, MA, USA, #25200056). Cells were pelleted by centrifugation at 300 g for 10 min at room temperature, resuspended in fresh culture medium and transferred to new culture flasks. Cell lines were cryopreserved in FBS supplemented with 10% dimethyl sulfoxide (DMSO, Sigma-Aldrich, St. Louis, MO, USA; #D2650).

### 4.2. Cell Treatments

One day prior to treatment, 100,000 cells per well were seeded in triplicates in 6-well plates (Fischer Scientific, Waltham, MA, USA, #140675). On the day of treatment, medium was replaced with fresh medium containing the specified stimulation agents. Cells were treated with either 30 ng/mL human IFN-γ (Miltenyi, Bergisch Gladbach, Germany, #130-096-872), except for MCF-7 cells that were treated with 24 ng/mL IFN-γ, diluted in deionized water containing 0.1% bovine serum albumin (BSA; Sigma-Aldrich, St. Louis, MO, USA, #A7030) or 10 μM and 100 μM 5-aza-dC (Calbiochem, Sigma-Aldrich, St. Louis, MO, USA, #189826-10MG) diluted in DMSO or both 30 ng/mL human IFN-γ and 10 μM 5-aza-dC. Stimulation with deionized water containing 0.1% BSA or DMSO added in the same concentrations as for the respective assays was used as control. All assays were performed in the same cell passage. Cells treated with IFN-γ were incubated for two days and cells treated with 5-aza-dC were incubated for three and six days until analyzed by flow cytometry and harvested for DNA and RNA purification. Cells treated with 5-aza-dC for 6 days had 2 mL medium removed and replaced with fresh medium without 5-aza-dC on day 3.

### 4.3. Flow Cytometry

Untreated and treated cells were harvested and analyzed by flow cytometry. All washing and staining of the cells were performed with BD FACSFlow^TM^ buffer (BD BioSciences, Franklin Lakes, NJ, USA) or PBS supplemented with 1–10% FBS. To discriminate live and dead cells, LIVE/DEAD Fixable Violet Dead Cell Stain Kit (Life Technologies, Carlsbad, CA, USA, #L34955) was used according to the manufacturer’s protocol. Afterwards, cells were washed once, pelleted by centrifugation and resuspended in 100 μL buffer. Cells were stained for 15 min with antibodies PE anti-human HLA-G (clone: 87G;Exbio, Prague, CZ, #1P-437-C100), PerCP-Cy5.5 anti-human HLA-A2 (clone: BB7.2; Biolegend, San Diego, CA, USA, #343316), PE-Cy7 anti-human HLA-E (clone: 3D12; Biolegend, San Diego, CA, USA, #342608), APC anti-human HLA Class I Bw6 (clone: REA143; Miltenyi, Bergisch Gladbach, Germany, #130-099-845), APC-eFluor 780 anti-human HLA-DR (clone: LN3; Thermofisher, Waltham, MA, USA, #47-9956-42) and BV510 anti-human HLA-C (clone: DT9; BD Biosciences, Franklin Lakes, NJ, USA #747597). The cells were washed once in 3 mL buffer, resuspended in 200 μL buffer and immediately analyzed on a FACSCanto II flow cytometer (BD BioSciences, Franklin Lakes, NJ, USA) using FACS Diva Software (BD BioSciences, Franklin Lakes, NJ, USA, version 8.01). For each sample, 50,000 events were recorded. Further analysis was performed using FACS Diva Software and FlowJo^TM^ Software (BD Biosciences, Franklin Lakes, NJ, USA, version 10).

### 4.4. DNA and RNA Extraction and cDNA Synthesis

For DNA and RNA purification, cell pellets were harvested as described above. The harvested cells were quickly stored as a dry pellet at −80 °C. Genomic DNA and total RNA were extracted simultaneously using the AllPrep DNA/RNA Mini Kit (Qiagen, Hilden, Germany, #80204) following the manufacturer’s protocol for *Simultaneous Purification of Genomic DNA and Total RNA from Animal Cells*. Cells were lysed and homogenized by vortexing. Purification of RNA alone was performed using the RNeasy^®^ Micro Kit (Qiagen, Hilden, Germany, #74004) following manufacturer’s protocol for *Purification of Total RNA from Animal and Human cells*. DNA was eluted in 50 μL elution buffer supplied with the kit and RNA was eluted in 30 μL RNase-free water supplied with the kits. DNA and RNA concentrations were measured using DropSense 96 (Unchained Labs, Pleasanton, CA, USA). The DNA was stored at −20 °C until use. The total RNA was stored at −80 °C until use. Quality of the purified RNA was assessed from a selection of the samples by automated electrophoresis using the Agilent 2100 Bioanalyzer system (Agilent, Santa Clara, CA, USA) and the Agilent RNA 6000 Pico Kit (Agilent, Santa Clara, CA, USA, #5067-1513) following manufacturer’s instructions. First-strand cDNA synthesis with SuperScript^®^ VILO™ cDNA synthesis kit (Invitrogen, ThermoFisher, Waltham, MA, USA, #11754050) was performed using maximum 2.5 μg of the extracted RNA and following the manufacturer’s instructions, and cDNA was stored at −20 °C until further use.

### 4.5. Digital Droplet PCR

Droplet digital (dd)PCR was performed using the QX100 Droplet Digital PCR system (BioRad, Hercules, CA, USA) as previously described [32]. In short, cDNA equivalent to 70–250 ng of total RNA, 10 μL 2x ddPCR^TM^ Supermix for Probes (Bio-Rad, Hercules, CA, USA, #186-3024), 10 μM of HLA-G forward primer (5′-AGC AGT CTT CCC TGC CCA CC-3′) and HLA-G reverse primer (5′-AGC TCC CTC CTT TTC AAT CTG AGC TC-3′), 4 μM probe (5′-FAM-CCT TGC AGC TGT AGT CAC TGG AGC TGC GGT CG-BHQ-1-3′) and nuclease-free water in a total volume of 20 μL were used for each reaction. Cycling conditions were: 95 °C for 10 min, followed by 40 cycles of 95 °C for 15 s, 62 °C for 1 min, 72 °C for 1 min, finally ending reaction at 98 °C for 10 min and hold at 4 °C. Specific amplicons were confirmed by electrophoresis on a 2% agarose gel. Results were analyzed using the QuantaSoft^®^ software version 1.3.2.0 (Bio-Rad, Hercules, CA, USA).

### 4.6. PCR and Fragment Analysis

PCR was performed with 90–320 ng cDNA, using 13.5 μL of GoTaq^®^ Hot Start Master Mix (Promega, Madison, WIS, USA, #M5133), 1 μM of HLA-G1-6 forward primer (5′-CAC AGA CTG ACA GAA TGA ACC TGC A-3′) and HLA-G1-6 reverse primer (5′-FAM-GGA AGG AAT GCA GTT CAG CAT GA-3′), and nuclease-free water in a total volume of 25 μL for each reaction.. The PCR was run under following conditions: 95 °C for 2 min, 29 cycles of 95 °C for 30 s, 62 °C for 2 min and 72 °C for 2 min, followed by 72 °C for 10 min and ending with hold at 4 °C. The PCR product was used directly for fragment analysis or stored overnight in the dark at 4 °C. For fragment analysis, 10 μL of nuclease-free water and 0.5 μL of GeneScan^TM^ 1200 LIZ^TM^ dye size standard (Life Technologies, Carlsbad, CA, USA, #4379950) were added to each well containing 2 μL of the PCR product and subsequently denatured at 95 °C for 3 min. followed by incubation on ice for 2 min. Fragment analysis on the ABI3500 Genetic Analyzer (Applied Biosystems, Waltham, MA, USA) was performed after the cooling. The PCR fragment lengths were analyzed using the GeneMarker^®^ software (SoftGenetics, PA, USA). Analysis on 10% of randomly chosen samples were performed in duplicates.

### 4.7. Genotyping of the HLA-G 14 bp Ins/Del Polymorphism

Genotyping of the *HLA-G* 14 bp ins/del polymorphism in JEG-3, FM-55M2, FM-56, MDA-MB-231 and MCF-7 cells was performed by TaqMan assay as previously described [68]. Briefly, 200-250 ng DNA, 10 μL 2x LightCycler 480 mastermix for probes (Roche Diagnostics, Basel, CH), 0.5 μM of HLAG14 forward primer (5′-GTG ATG GGC TGT TTA AAG TGT CAC C-3′) and HLAG14 reverse primer (5′-GGA AGG AAT GCA GTT CAG CAT GA-3′), 0.07 μM HLAG14 FAM probe (5′-FAM-CAA GAT TTG TTC ATG CCT TCC C-BHQ-1-3′), 0.3 μM HLAGdel Cy5 probe (5′Cy5-GAG TGG CAA GTC CCT TTG TG-BHQ-2-3′) and nuclease-free water in a total volume of 20 μL was used for each reaction. The real-time TaqMan PCR assay was performed using a LightCycler 480 instrument (Roche Diagnostics, Basel, CH). Cycling conditions were: 95 °C for 10 min, followed by 50 cycles of 95 °C for 10 s, 60 °C for 50 s, 72 °C for 1 s finally ending reaction by hold at 4 °C for 10 s.

### 4.8. Bisulfite Sequencing

Bisulfite conversion of 300–350 ng purified genomic DNA was carried out using the EZ DNA Methylation-Lightning Kit (Zymo Research, Nordic Biosite, Täby, SE, #D5031) following the manufacturer’s protocol. The *HLA-G* sequence was obtained from NCBI Reference Sequence NG_029039.1 and prediction of the 5′ CpG island from the UCSC Genome Browser. Target-specific bisulfite sequencing PCR primers with M13 tags were designed in three pairs overlapping and covering the CpG island. Pair F1R2: forward 5′-TTA GGT GAT AGG TTT TTA GAG AAG TTA ATT-3′, reverse 5′-ATT CCA TAT CTC CTC TTC CCA ATA-3′; pair F2R3: forward 5′-TTT AGG TTT TTA TTT TAT GAG GTA TTT TAG-3′, reverse 5′-ACC AAT CAT CCA CTA AAA AAT ATA AAA AC-3′; pair F3R1: 5′-AGA TTT TTT ATT TGG GAG AAT TTT AAG G-3′, reverse 5′-ATA TTA ATC CCA TTT TCC TCC TCT CC-3′. Specificity of bisulfite-specific primers were confirmed using the online tool BiSearch [69,70]. In brief, the PCR was performed with 2 μL bisulfite converted DNA, 5 μL 2x KAPA HiFi HotStart Uracil+ ReadyMix (Roche Diagnostics, Basel, CH, #7959052001) and 6 μM of each primer in a total volume of 10 μL for each reaction. A touchdown PCR for bisulfite converted DNA was run under the following conditions: 95 °C for 5 min., 17 cycles of: 95 °C for 20 s, 65 °C for 20 s and 72 °C for 45 s, followed by 30 cycles of: 95 °C for 20 s, 50 °C for 20 s and 72 °C for 45 s, finally ending reaction at 72 °C for 5 min and hold at 4 °C. PCR products were purified using AMPureXP on Biomek 4000 (both from Beckman Coulter, Brea, CA, USA). Sanger sequencing was performed using the BigDye^TM^ Terminator v3.1 Cycle Sequencing Kit (Applied Biosystems, Waltham, MA, USA, #4337455), 2 μL PCR product, 4 μM sequencing primer and nuclease-free water in a total volume of 10 μL for each reaction. The sequencing reaction was carried out at the following conditions: 96 °C for 1 min, 35 cycles of 96 °C for 30 s, 50 °C for 15 s, 60 °C for 4 min, finally ending reaction by hold at 4 °C. The products were purified using CleanSeq (Beckman Coulter, Brea, CA, USA) on Biomek 4000. Bidirectional Sanger sequencing was performed on ABI3500 Genetic Analyzer (Applied Biosystems, Waltham, MA, USA). The results were analyzed using Mutation Surveyor (SoftGenetics, PA, USA).

### 4.9. Statistical Analysis

If not otherwise stated, experiments were performed in triplicates. Figures and statistical analysis were executed using GraphPad Prism^®^ software (GraphPad, San Diego, CA, USA). *p*-values < 0.05 were considered statistically significant, and marked in the figures accordingly: * *p* < 0.05; ** *p* < 0.01; *** *p* < 0.001 and **** *p* < 0.0001. Student’s unpaired *t*-test with Welch’s correction was used for comparison of IFN-γ and 5-aza-dC treated samples to their respective controls for flow cytometry data and ddPCR results.

## Figures and Tables

**Table 1:** *HLA-G* 14 bp insertion/deletion (ins/del) 3′UTR polymorphism in the cancer cell lines.

Cell Line	14 bp Polymorphism
MDA-MB-231	del/del
MCF-7	del/del
FM-55M2	ins/del
FM-56	ins/ins
JEG-3	ins/ins

**Table 2:** Summary of findings.

Cell Line	Condition	HLA-A	HLA-B	HLA-C	HLA-E	HLA-G *	HLA-DR
MDA-MB-231	Untreated **	+	+	(+) ***	(+)	−	−
	IFN-γ	↑	↑	↑	↑	−	↑
	5-Aza-dC						
	10 µM, day 3	↑	↑	−	−	↑	↑
	10 µM, day 6	↑	↑	↑	↑	-	↑
	100 µM, day 3	↑	↑	↑	↓	↑	↑
	100 µM, day 6	−	↑	↑	↑	−	↑
MCF-7	Untreated	+	−	−	(+)	−	−
	IFN-γ	↑	↑	−	−	−	−
	5-Aza-dC						
	10 µM, day 3	↑	↑	↑	-	↑	−
	10 µM, day 6	↑	↑	↑	↑	↑	−
	100 µM, day 3	−	−	−	−	↑	−
	100 µM, day 6	↑	↑	↑	-	↑	-
FM-55M2	Untreated	+	+	−	(+)	−	(+)
	IFN-γ	↑	↑	↑	↑	−	↑
	5-Aza-dC						
	100 µM, day 3	↓	↑	-	-	↑	−
	100 µM, day 6	↓	−	↓	↑	−	↑
FM-56	Untreated	−	(+)	−	−	−	+
	IFN-γ	−	↑	↑	↑	−	↑
	5-Aza-dC						
	100 µM, day 3	−	↑	−	−	↑	−
	100 µM, day 6	↑	↑	↑	↑	↑	−
JEG-3	Untreated	(+)	(+)	+	+	+	−

* Combined results for HLA-G surface expression and mRNA expression. ** Expression based upon comparison between the specific marker and isotype control. *** Parentheses indicate possible low expression or expression difficult to interpret because of differences in the MFI distributions of isotype and specific marker antibody, respectively.

## Supplementary Materials

Supplementary materials can be found at https://www.mdpi.com/1422-0067/21/12/4307/s1. Figure S1: Representative histograms for breast cancer cell lines showing the fluorescence intensity of HLA molecules before and after treatment, Figure S2: Representative histograms for malignant melanoma cell lines showing the fluorescence intensity of HLA molecules before and after treatment and for JEG-3 cells, Figure S3: HLA surface expression on malignant melanoma cells stimulated with IFN-γ.
