# Supplementary material for: Characterization of HLA-G Regulation and HLA Expression in Breast Cancer and Malignant Melanoma Cell Lines upon IFN-γ Stimulation and Inhibition of DNA Methylation
Source: Int J Mol Sci. 2020 Jun 17;21(12):4307. doi: 10.3390/ijms21124307 (PMC7352735; doi:10.3390/ijms21124307)
Supplement: Supplementary file 1 [file ijms-21-04307-s001.pdf]

Supplementary Figures

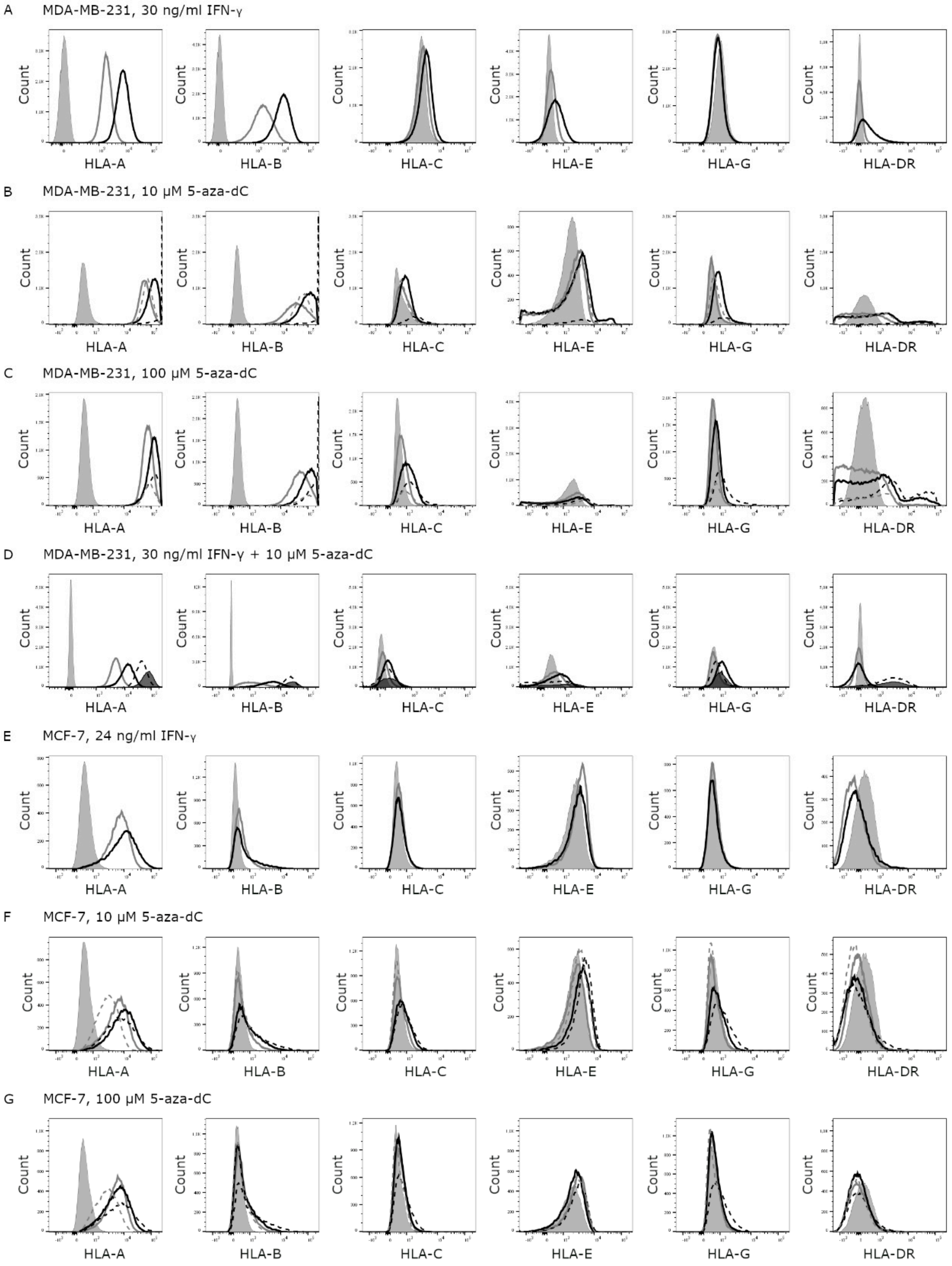

Figure S1. Representative histograms for breast cancer cell lines showing the fluorescence intensity of HLA molecules before and after treatment. From left to right, the HLA proteins are HLA-A, HLA-B, HLA-C, HLA-E, HLA-G, and HLA-DR. (**A**,**B**,**C**,**D**) MDA-MB-231 cells treated with 30 ng/ml IFN-γ for two days (**A**), 10 µM 5-aza-dC for three and six days (**B**), 100 µM 5-aza-dC for three and six days (**C**) and with a combination of 30 ng/ml IFN-γ and 10 µM 5-aza-dC for three days (**D**). (**E**, **F**, **G**) MCF-7 cells treated with 24 ng/ml IFN-γ for two days (**E**), 10 µM 5-aza-dC for three and six days (**F**), and 100 µM 5-aza-dC for three and six days (**G**). (**A**-**C**, **E**-**G**) Filled grey: cells stained with isotype antibody as a negative control; solid grey: untreated control cells on day two/three; solid black: treated cells on day two/three; dashed grey: untreated control cells on day six; dashed

black: treated cells on day six. (**D**) Filled grey: cells stained with isotype antibody as a negative control; solid grey: untreated control cells; solid black: 5-aza-dC-treated cells; dashed black: IFN-γ-treated cells; filled black: Cells treated with IFN-γ and 5-aza-dC.

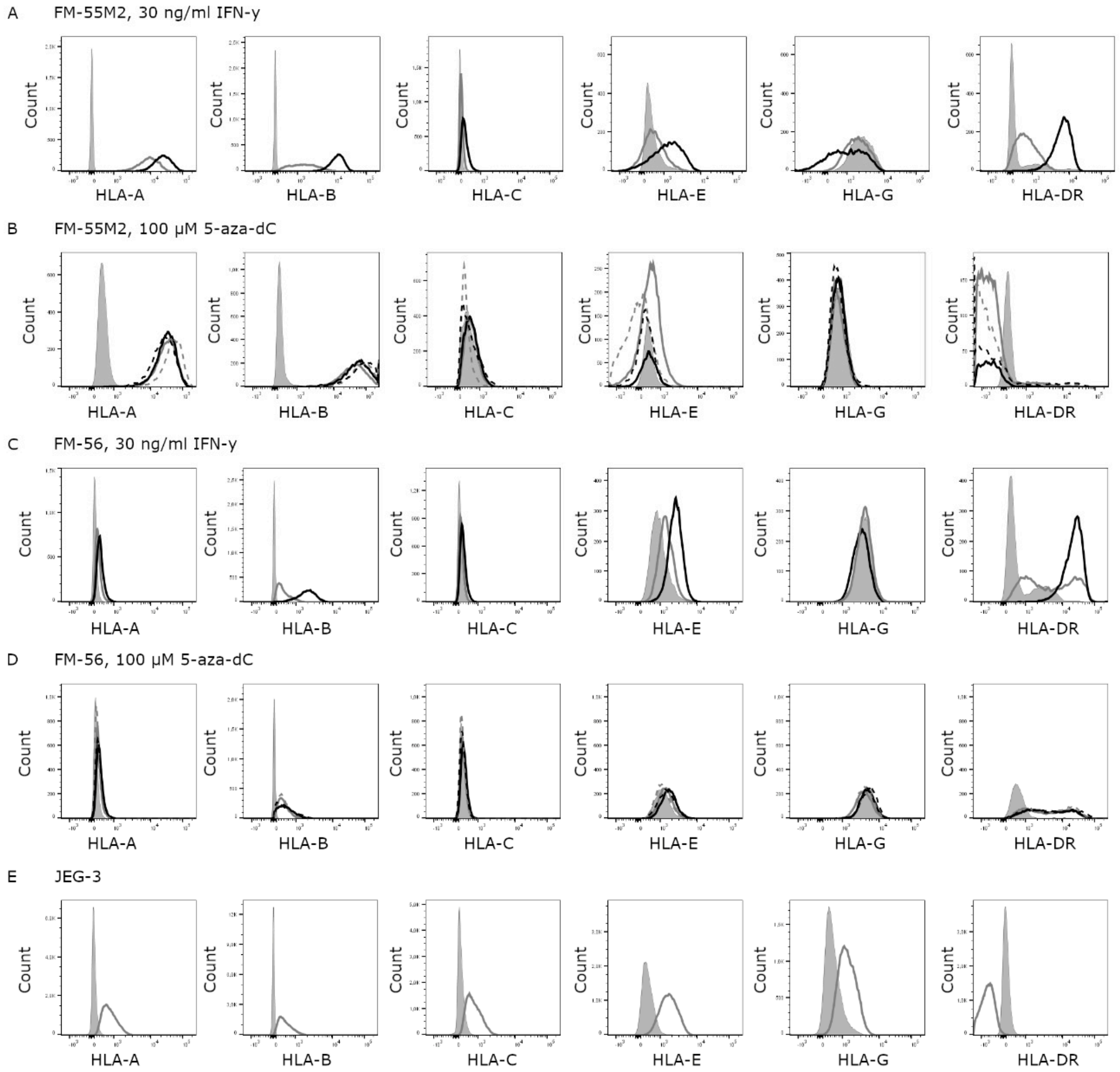

Figure S2. Representative histograms for malignant melanoma cell lines showing the fluorescence intensity of HLA molecules before and after treatment and for JEG-3 cells. From left to right, the HLA proteins are HLA-A, HLA-B, HLA-C, HLA-E, HLA-G, and HLA-DR. (**A**, **B**) FM-55M2 cells treated with 30 ng/ml IFN-γ for two days (**A**) and 100 μM 5-aza-dC for three and six days (**B**). (**C**, **D**) FM-56 cells treated with 30 ng/ml IFN-γ for two days (**C**) and 100 μM 5-aza-dC for three and six days (**D**). (**E**) Untreated JEG-3 cells as a positive control for HLA-G expression. Filled grey: cells stained with isotype antibody as a negative control; solid grey: untreated control cells on day two/three; solid black: treated cells on day two/three; dashed grey: untreated control cells on day six; dashed black: treated cells on day six.

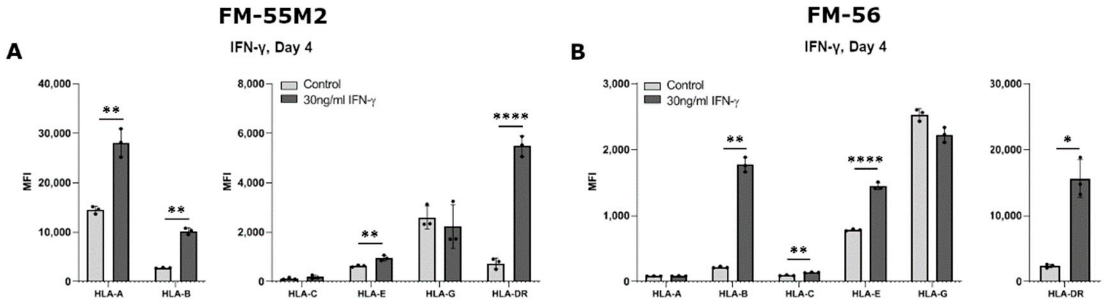

Figure S3. HLA surface expression on malignant melanoma cells stimulated with IFN-γ. Flow cytometry analysis of HLA-A, HLA-B, HLA-C, HLA-G, HLA-E, and HLA-DR expression. (**A**) FM-55M2 cells treated with 30 ng/ml IFN-γ for four days. (**B**) FM-56 cells treated with 30 ng/ml IFN-γ for four days. Stimulation of each cell line was performed as separate experiments. Shown are median fluorescence intensity (MFI) with mean ± SD, each dot represents one sample. * $p < 0.05$, ** $p < 0.01$, **** $p < 0.0001$ (Student's unpaired t-test with Welch's correction).
